# Gli1^+^ Cells Residing in Bone Sutures Respond to Mechanical Force via IP_3_R to Mediate Osteogenesis

**DOI:** 10.1155/2021/8138374

**Published:** 2021-08-12

**Authors:** Xiaoyao Huang, Zihan Li, Peisheng Liu, Meiling Wu, An-qi Liu, Chenghu Hu, Xuemei Liu, Hao Guo, Xiaoxue Yang, Xiaohe Guo, Bei Li, Xiaoning He, Kun Xuan, Yan Jin

**Affiliations:** ^1^State Key Laboratory of Military Stomatology & National Clinical Research Center for Oral Diseases & Shaanxi Clinical Research Center for Oral Diseases, Department of Preventive Dentistry, School of Stomatology, The Fourth Military Medical University, Xi'an 710032, China; ^2^State Key Laboratory of Military Stomatology & National Clinical Research Center for Oral Diseases & Shaanxi International Joint Research Center for Oral Diseases, Center for Tissue Engineering, School of Stomatology, The Fourth Military Medical University, Xi'an 710032, China; ^3^College of Life Science, Northwest University, Xi'an, China

## Abstract

Early orthodontic correction of skeletal malocclusion takes advantage of mechanical force to stimulate unclosed suture remodeling and to promote bone reconstruction; however, the underlying mechanisms remain largely unclear. Gli1^+^ cells in maxillofacial sutures have been shown to participate in maxillofacial bone development and damage repair. Nevertheless, it remains to be investigated whether these cells participate in mechanical force-induced bone remodeling during orthodontic treatment of skeletal malocclusion. In this study, rapid maxillary expansion (RME) mouse models and mechanical stretch loading cell models were established using two types of transgenic mice which are able to label Gli1^+^ cells, and we found that Gli1^+^ cells participated in mechanical force-induced osteogenesis both in vivo and in vitro. Besides, we found mechanical force-induced osteogenesis through inositol 1,4,5-trisphosphate receptor (IP_3_R), and we observed for the first time that inhibition of Gli1 suppressed an increase in mechanical force-induced IP3R overexpression, suggesting that Gli1^+^ cells participate in mechanical force-induced osteogenesis through IP_3_R. Taken together, this study is the first to demonstrate that Gli1^+^ cells in maxillofacial sutures are involved in mechanical force-induced bone formation through IP_3_R during orthodontic treatment of skeletal malocclusion. Furthermore, our results provide novel insights regarding the mechanism of orthodontic treatments of skeletal malocclusion.

## 1. Introduction

Skeletal malocclusion is the most severe type of malocclusion since it is difficult to correct by orthodontic methods in adults; however, skeletal malocclusion can be corrected using orthopedic force when the patient is at their growth peak with unclosed sutures [1]. It has been reported that unclosed maxillofacial sutures can be remodeled using orthopedic force, and maxillofacial sutures are the residence of mesenchymal stem cells (MSCs) which participate in maxillofacial bone development and damage repair [2, 3]. Moreover, MSCs are commonly known to tend to osteogenesis after stimulation by mechanical stretching or incubation on stiff culture substrates [4–7]. However, it remains largely unclear whether MSCs that reside in maxillofacial sutures participate in orthopedic force-induced bone remodeling during skeletal malocclusion correction. Furthermore, MSCs are not single-cell populations but constitute a complex mixture of multiple types of subpopulations [8]. Gli1^+^ cells, which are closely associated with osteogenesis and odontogenesis, are one of the subpopulations of MSCs [9], and they were reported to reside in the maxillofacial suture, the periosteum, and the nearby dura. When adjacent bone is injured, Gli1^+^ cells residing in the suture differentiate and participate in repair mechanisms [10, 11]. Our previous study found that Gli1^+^ cells in periodontal tissues are involved in orthodontic force-induced alveolar bone remodeling [12]; however, whether and how Gli1^+^ cells from maxillofacial sutures participate in orthopedic force, greater than orthodontic force, induced maxillofacial bone remodeling remains unknown.

Inositol 1,4,5-trisphosphate receptor (IP_3_R) is a channel which mainly located on the endoplasmic reticulum (ER) membrane controlling Ca^2+^ release from the ER [13, 14]. Applying mechanical force on cells causes deformation of cellular membrane structures, and membrane proteins such as IP_3_R can be easily affected by such force [15–17]. A previous study showed that mechanical stretching induced IP_3_R-mediated Ca^2+^ release from the ER and caused intracellular Ca^2+^ concentrations to increase [18]. The increase in intracellular Ca^2+^ concentrations was reported to improve osteogenic potential of mouse bone marrow mesenchymal stem cells (BMMSCs) [19]; however, it is unclear whether Gli1^+^ cells respond to mechanical force through IP_3_R-regulated intracellular Ca^2+^ concentration change.

This study is aimed at exploring the role of Gli1^+^ cells in mechanical force-induced bone remodeling and at elucidating the underlying mechanisms. We verified that Gli1^+^ cells residing in maxillofacial bone sutures are involved in mechanical force-induced maxillofacial bone formation. And IP_3_R, the calcium ion channel located on the ER, was involved in mechanical force-induced osteogenesis and was regulated by Gli1^+^ cells. In conclusion, our study revealed the mechanical sensor role of Gli1^+^ cells residing in maxillofacial bone sutures, and we describe for the first time that Gli1^+^ cells respond to mechanical force through IP_3_R-mediated intracellular Ca^2+^ concentration changes so as to regulate osteogenesis. Our findings thus provide novel insights into the mechanism of early orthodontic treatments of skeletal malocclusion.

## 2. Materials and Methods

### 2.1. Study Animals

We used the mouse strains *Gli1-LacZ* (JAX no. 008211), *Gli1CreERT2* (JAX no. 007913), and *ROSA26-mT/mG* (JAX no. 007676) acquired from the Jackson Laboratory. The mice were housed in a specific pathogen-free environment under a 12 h light cycle. At an age of 4 weeks, genotyping was performed by PCR according to the procedure recommended by the Jackson Laboratory (primers are showed in Table 1), and mice with suitable genotypes were used for subsequent experiments at an age of 6–8 weeks. All experiments involving animals were performed according to the guidelines of the Intramural Animal Use and Care Committee of the Fourth Military Medical University, Xi'an, China (approval number: 2020-kq-006).

### 2.2. Animal Treatments

**Figure 1:**
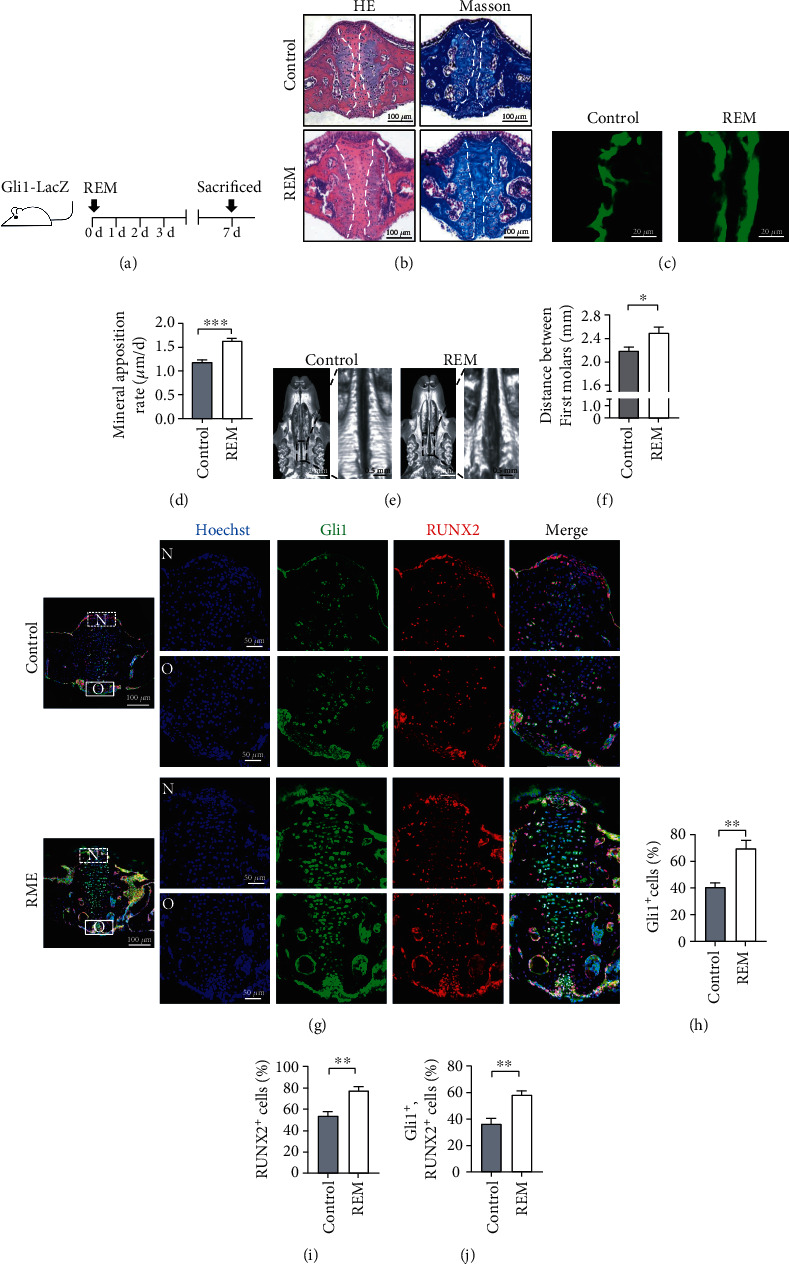
Gli1^+^ cells residing in palatal sutures involved in mechanical force-induced maxillofacial bone remodeling. (a) Experimental procedure: *Gli1-LacZ* mice were sacrificed seven days after rapid maxillary expansion (RME) treatment. Control mice were not subjected to RME and were sacrificed at the same time. (b) Coronal sections of maxillae of RME and control mice were stained with hematoxylin/eosin and Masson's staining. Compared with that in the controls, maxillofacial sutures in RME mice were expanded, and cells residing on the nasal and oral sides of the suture had increased. Collagen in the suture was rearranged along the direction of the mechanical force. The area between two dotted lines shows the midpalatal suture. Scale bar: 100 *μ*m; *n* = 3. (c) New bone formation was visualized using calcein; the distance between green fluorescence lines indicates newly formed bone. Scale bar: 20 *μ*m; *n* = 3. (d) New bone deposition rate was faster in the RME group, ^∗∗∗^*P* < 0.005; *n* = 3. (e) Micro-computed tomography (micro-CT) shows horizontal plane views of the maxillae. Scale bar: 2 mm. The left panel shows magnified sutures in the boxes. Scale bar: 0.5 mm. (f) The distance between two first molars was widen in RME mice. ^∗^*P* < 0.05; *n* = 3. (g) Immunofluorescence staining presents Gli1 (green) and RUNX2 (red) expression in the midpalatal suture areas of the RME group and control group. Scale bar: 100 *μ*m. Regions in boxes are magnified in the right panel: “N” indicates the nasal side of the midpalatal suture and “O” indicates the oral side. Scale bar: 50 *μ*m. (h) Proportions of Gli1^+^ cells increased on nasal and oral sides and midpalatal suture in RME mice. ^∗∗^*P* < 0.01; *n* = 3. (i) Proportion of RUNX2^+^ cells in midpalatal suture increased in RME mice. ^∗∗^*P* < 0.01; *n* = 3. (j) Gli1^+^ and RUNX2^+^ cells increased in sutures of RME mice. ^∗∗^*P* < 0.01; *n* = 3.

**Figure 2:**
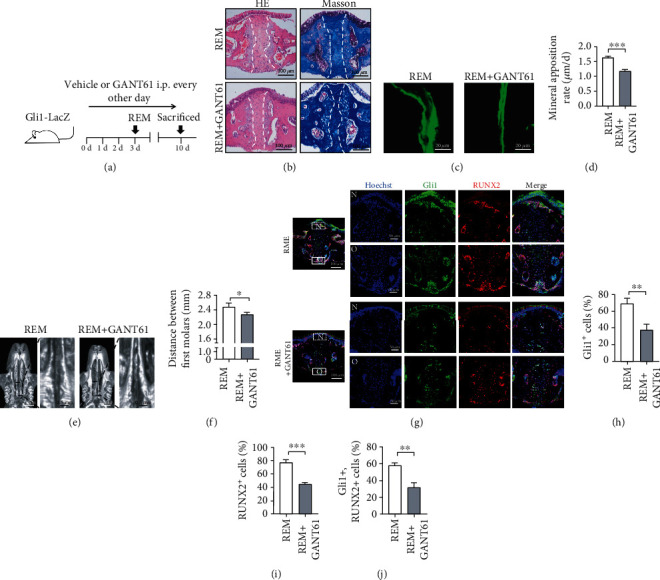
Pharmacological inhibition of Gli1^+^ cells suppresses mechanical force-induced maxillofacial bone remodeling. (a) Experimental procedure: *Gli1-LacZ* mice were treated with GANT61 every second day before they were sacrificed, and RME was induced on day 3 (RME+GANT61). Mice in the RME group were treated with vehicle. (b) HE and Masson's staining showed decreased midpalatal suture remodeling in GANT61-treated mice. The midpalatal suture is shown between the two dotted lines. Scale bar: 100 *μ*m; *n* = 3. (c) Calcein labelling shows that new bone formation is less in the RME+GANT61 group. Scale bar: 20 *μ*m; *n* = 3. (d) New bone deposition rate decreased in the RME+GANT61 group, ^∗∗∗^*P* < 0.005; *n* = 3. (e) Micro-CT shows the horizontal plane views of maxillae of both groups. Scale bar: 2 mm. The left panel shows magnified sutures (in boxes), and GANT61-treated mice show narrow midpalatal sutures. Scale bar: 0.5 mm. (f) The distance between two first molars decreased in GANT61 application mice. ^∗^*P* < 0.05; *n* = 3. (g) Gli1 (green) and RUNX2 (red) expression in the midpalatal suture areas of both groups visualized by immunofluorescence staining. Scale bar: 100 *μ*m. Regions in boxes are magnified in the right panel: “N” indicates the nasal side of the midpalatal suture and “O” indicates the oral side. Scale bar: 50 *μ*m. (h) Proportions of Gli1^+^ cells decreased in the nasal and oral sides and in the midpalatal suture in GANT61-treated mice. ^∗∗^*P* < 0.01; *n* = 3. (i) The proportions of RUNX2^+^ cells in the midpalatal suture decrease in the RME+GANT61 group. ^∗∗∗^*P* < 0.005; *n* = 3. (j) Gli1^+^ and RUNX2^+^ cells decreased in the sutures of the RME+GANT61 group. ^∗∗^*P* < 0.01; *n* = 3.

Eight-week-old transgenic *Gli1-LacZ* mice were assigned to three groups (RME, RME+GANT61, and control) with three individuals per group. The RME and RME+GANT61 groups were treated as shown in Figure 1(a) and Figure 2(a), respectively, and control mice were not subjected to the RME treatment. The RME model was established as previously described [20]. Briefly, using 0.014-inch Australian arch wire, an opening loop was placed at the palatal side of the molars of the upper jaw and was fixed using a light-cured adhesive (3M Unitek, Monrovia, CA, USA). The force applied on the palatal suture was approximately 0.56 N.

### 2.3. Drug Administration

**Figure 3:**
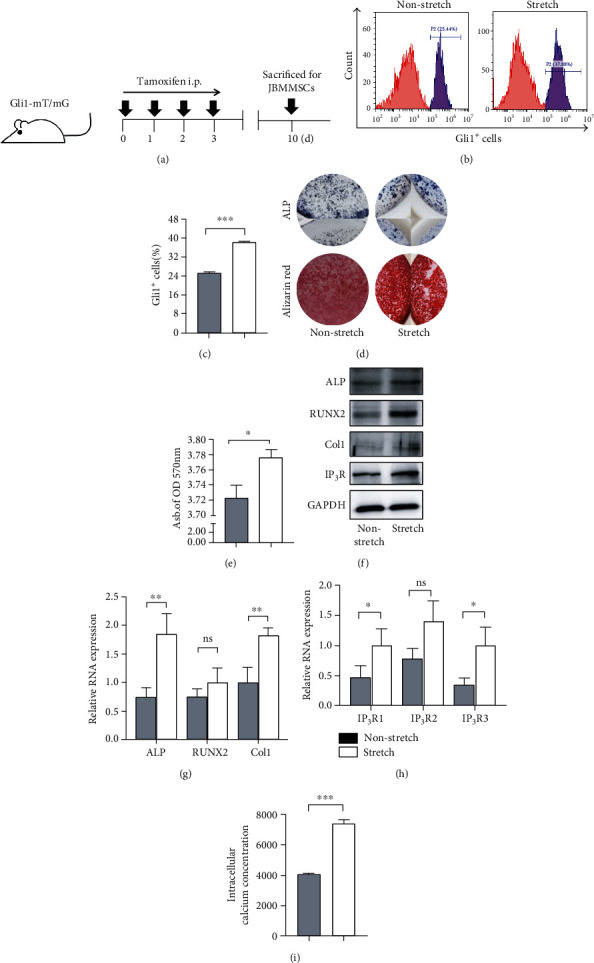
Mechanical stretching induces Gli1^+^ cell increases and regulates JBMMSC osteogenesis via IP_3_R-mediated intracellular calcium increases. (a) Experimental procedure: *Gli1-mT/mG* mice were treated with tamoxifen for 4 consecutive days and were sacrificed after 7 days to isolate JBMMSCs. (b) Flow cytometry analysis of the proportion of Gli1^+^ cells in JBMMSCs; the stretch-treated group showed an increase in Gli1^+^ cells; *n* = 3. (c) The proportion of Gli1^+^ cells was higher in the stretch group. *P* < 0.005; *n* = 3. (d) Alkaline phosphatase (ALP) activity of JBMMSCs subjected to mechanical stretching was higher after osteogenic induction for seven days. Mineralized nodules formed by JBMSCs were detected using alizarin red staining after osteogenic induction for 14 days. (e) The abundance of mineralized nodules in the stretch group was higher. ^∗^*P* < 0.05; *n* = 3. (f) Western blot analysis of ALP, RUNX2, Col1, and IP_3_R protein expression levels in JBMSCs which after mechanical stretching were upregulated, compared with control cells; *n* = 3. (g) RT-qPCR showed upregulation of ALP, RUNX2, and Col1 RNA expression in JBMSCs after mechanical stretching, compared with control cells. ^∗∗^*P* < 0.01; ^ns^*P* > 0.05; *n* = 3. (h) RT-qPCR showed upregulation of IP_3_R1, IP_3_R2, and IP_3_R3 RNA expression in JBMSCs after mechanical stretching, compared with control cells. ^∗^*P* < 0.05; ^ns^*P* > 0.05; *n* = 3. (i) Intracellular calcium concentration visualized at mean fluorescence intensity was increased in the stretch group. ^∗∗∗^*P* < 0.005; *n* = 3.

To inhibit Gli1 expression, GANT61 (HY-13901; MedChemExpress, South Brunswick, NJ, USA) was dissolved according to the manufacturer's instructions and was intraperitoneally injected at 40 mg/kg of body weight every second day until the mice were sacrificed. For in vitro experiments, 10 *μ*M GANT61 was administered for approximately 6 days after incubation of primary cells. To induce Cre activity, tamoxifen (T5648; Sigma-Aldrich, St. Louis, MO, USA) was intraperitoneally injected at 100 mg/g of body weight for 4 consecutive days. Follow-up experiments were performed 7 days after this treatment (Figure 3(a)). Alizarin complexone (A3882; Sigma-Aldrich) and calcein (C0875; Sigma-Aldrich) used to label newly formed bone were intraperitoneally injected at 100 mg/g of body weight on the day before rapid maxillary expansion (RME) and sacrificing, respectively. To inhibit the function of IP_3_R, 2-aminoethyl diphenylborinate (2-APB; 3170846; Merck Millipore, Darmstadt, Germany) was applied at a concentration of 70 *μ*M, 24 h after incubation.

### 2.4. Hematoxylin/Eosin (HE) and Masson's Staining

Freshly collected maxillae were fixed at 4°C overnight using 4% paraformaldehyde (Sigma-Aldrich). Then, 17% ethylenediaminetetraacetic acid solution (MP Biomedicals, Santa Ana, CA, USA) was used to decalcify the samples at 4°C. Decalcified samples were embedded in paraffin and were cut into 4 *μ*m thick sections along the coronal plane. HE (Leica, Wetzlar, Germany) staining and Masson's staining (Baso, Zhuhai, China) were performed according to the manufacturer's instructions.

### 2.5. Micro-Computed Tomography (Micro-CT) Analysis and Calcein Staining

Maxillae were collected, fixed in 4% paraformaldehyde, and scanned by micro-CT (Siemens Inveon, Erlangen, Germany). The horizontal plane was examined, and distances between the bilateral first molars were measured using ImageJ (Media Cybernetics, USA). For calcein staining, fixed samples were dehydrated and embedded in resin. Samples were then cut along the coronal plane and were observed by laser confocal microscopy (A1 Plus, Nikon, Tokyo, Japan), and the distances between two green lines were measured using ImageJ.

### 2.6. Immunofluorescence Staining

Frozen sections of decalcified samples were used for immunofluorescence staining. Briefly, sections were permeabilized at room temperature (RT) for 10 min using 0.3% TritonX-100 (Sigma-Aldrich); they were blocked using goat serum (Sigma-Aldrich) at 37°C for 30 min and were then incubated with primary antibodies (Table 2) at 4°C overnight. On the following day, the samples were incubated with the respective secondary antibodies in the dark for 2 h at 37°C, and nuclei were dyed using Hoechst (MedChemExpress) for 15 minutes at RT.

### 2.7. Isolation of JBMMSCs

Jaw bones of six-week-old tamoxifen-treated *Gli1-mT/mG* transgenic mice were separated and were washed using phosphate-buffered saline (PBS; Gibco, Thermo Fisher Scientific, Waltham, MA, USA). All teeth were carefully removed, and residual bone was cut to pieces using sterile scissors. Bone tissue was then digested using dispase II (Sigma-Aldrich) and collagenase I (MP Biomedicals) at a proportion of 1 : 1 for 1 h at 37°C. Sufficient culture medium containing 20% fetal bovine serum (FBS; Tianhang, Zhejiang, China) was added to terminate digestion after which the mixture was centrifuged at 800 rpm for 5 min. The supernatant was removed, and the pellet was resuspended in culture medium containing *α*-minimal essential medium (*α*-MEM; Invitrogen, Thermo Fisher Scientific, Waltham, MA, USA), 20% FBS, 2 mM L-glutamine (Invitrogen), 100 U/mL penicillin (Invitrogen), and 100 g/mL streptomycin (Invitrogen) after which it was incubated at 37°C and 5% CO_2_. Passage 3 was used for the following experiments.

### 2.8. Mechanical Stretching of JBMMSCs

JBMMSCs were seeded on six-well BioFlex culture plates (Flexcell, Burlington, NC, USA) at a density of 2 × 10^5^ cells per well. When cells reached 80% confluence, 8% elongation at 0.5 Hz was applied for 6 h each day using a FX-4000™ Tension System (Flexcell). After 3 days, cells were harvested for the following experiments.

### 2.9. Alizarin Red and Alkaline Phosphatase (ALP) Staining

JBMMSCs were seeded in six-well plates at a density of 2 × 10^5^ cells per well, and when cells reached 70% confluence, the culture medium was replaced with osteogenic medium containing 50 *μ*g/mL ascorbic acid (MP Biomedicals), 2 mM *β*-glycerophosphate (Sigma-Aldrich), and 10 nM dexamethasone (Sigma-Aldrich). After 7 days of osteogenic induction, ALP staining was performed using an alkaline phosphatase assay kit (P0321; Beyotime, Shanghai, China) according to the manufacturer's protocol to test ALP activity. After 14 days, alizarin red staining was performed to assess the extent of mineralization. Briefly, cells were fixed using 4% paraformaldehyde for 30 min, washed using PBS, stained with alizarin red solution for 30 min, and then washed again using PBS. After recording pictures of alizarin red-stained cells, calcified nodules were dissolved using 10% cetylpyridinium chloride, and absorbance value was measured at 570 nm to assess calcium concentrations.

### 2.10. Oil Red O Staining

JBMMSCs were seeded in six-well plates at a density of 2 × 10^5^ cells per well. When the cells reached 70% confluence, the culture medium was replaced by adipogenic induction medium containing 0.5 mM 3-isobutyl-1-methylxanthine, 1 *μ*M dexamethasone, and 0.1 mM indomethacin (Sigma-Aldrich). After the 7-day adipogenic induction, cells were fixed using 4% paraformaldehyde for 30 min, washed using PBS, stained with Oil Red O (Aladdin, Shanghai, China) solution for 30 min, and then washed again using PBS.

### 2.11. Colony Formation Assay

Primary cells were used for the colony formation assay. Approximately 7 days after primary cell incubation, colonies were observed using a microscope. Cells were then fixed using 4% paraformaldehyde for 30 min, washed with PBS, stained with crystal violet solution for 30 min, and then washed again using PBS.

### 2.12. Flow Cytometry Analysis

Flow cytometry was used to identify JBMMSCs, to assess the proportion of Gli1^+^ cells in the complete JBMMSC population, and to measure concentrations of intracellular calcium ions. To identify JBMMSCs, cells were digested using 0.25% trypsin (MP Biomedicals) and were washed using PBS, after which the cells were resuspended in centrifuge tubes using 100 *μ*L PBS and incubated at RT for 45 min with 1 *μ*L primary antibodies per tube (Table 1). After washing twice using PBS, cells were examined by flow cytometry (FACSAria, BD Biosciences, San Jose, CA, USA). To assess the proportion of Gli1^+^ cells, the cells were digested using trypsin, washed using PBS, and subsequently analyzed by flow cytometry using the FITC channel. To measure intracellular calcium ion concentrations, cells were washed using PBS and were then incubated with 5 *μ*M Fluo-8AM (21081; AAT Bioquest, Sunnyvale, CA, USA) for 30 min at RT. After washing twice using PBS, cells were digested using trypsin and were examined by flow cytometry, and mean fluorescence intensity was used to evaluate intracellular calcium ion concentrations.

### 2.13. Reverse Transcription-Quantitative Polymerase Chain Reaction (RT-qPCR)

Total RNA was extracted from the samples using TRIzol reagent (Invitrogen), and RNA was reverse-transcribed to cDNA. Expression of IP_3_Rs and of osteogenic markers including Col1, ALP, and RUNX2 was evaluated using RT-qPCR as previously described [21], with GAPDH as an internal reference. PCR primer sequences are shown in Table 3.

### 2.14. Western Blotting

Total protein was extracted from the samples using lysis buffer (Beyotime), and protein concentrations were measured using a bicinchoninic acid (BCA) protein assay kit (Tiangen, Beijing, China) according to the manufacturer's instructions. Subsequently, 20 *μ*g total protein of each sample was separated by 10% sodium dodecyl sulfate-polyacrylamide gel electrophoresis (SDS-PAGE) and was transferred to a polyvinylidene fluoride (PVDF) membrane. After 2 h, membranes were blocked using 5% nonfat milk for 1 h and were then incubated with primary antibodies (Table 2) overnight at 4°C. On the following day, the membranes were incubated with the respective horseradish peroxidase-conjugated secondary antibodies for 2 h at RT. A chemiluminescent detection system was used to produce the readings.

### 2.15. Statistical Analyses

Data are shown as the means ± standard deviation. Analyses were performed using GraphPad Prism (version 8.0.0 for Windows, GraphPad Software, San Diego, CA, USA). Differences between two groups were tested using two-tailed unpaired Student's *t*-test, and statistical significance is reported at *P* < 0.05.

## 3. Results and Discussion

### 3.1. Gli1^+^ Cells Residing in Palatal Sutures Involved in Mechanical Force-Induced Maxillofacial Bone Remodeling

In order to investigate the role of Gli1^+^ cells in mechanical force-induced maxillofacial bone remodeling, we established a RME mouse model to imitate the procedure of orthodontic treatment of skeletal malocclusion using *Gli1-LacZ* transgenic mice to help detect Gli1^+^ cells after LacZ staining; as a control, we used mice that were not subjected to RME treatments (Supplementary Figure 1). HE staining showed that palatal sutures of mice in the experimental group were expanded and considerably wider than those of control mice (Figure 1(b)). In addition, periosteal cells on the nasal and oral sides of the palatal sutures increased and migrated into the palatal suture at the early stage of RME; however, cells on both sides decreased during the late stage (Supplementary Figure 2(a)). Masson's staining showed that collagen in the palatal suture was reoriented due to the effects of mechanical force (Figure 1(b)). Furthermore, dynamic bone labelling by calcein staining demonstrated that bone deposition in midpalatal of the RME group was much more than in the controls (Figures 1(c) and 1(d)). Moreover, micro-CT showed that the width of midpalatal suture was wider in the RME group (Figures 1(e) and 1(f)), indicating that palatal sutures of the RME group were expanded. To assess expression patterns in Gli1^+^ cells, we measured Gli1 expression using immunofluorescence staining and found that Gli1^+^ cells started to increase on the nasal and oral sides of the palatal suture to a maximum on day 7 after which they decreased over the following days (Figures 1(g) and 1(h); Supplementary Figure 2(b)). Mice of both groups were thus sacrificed on day 7 to conduct the follow-up experiments. In order to assess bone formation during RME, expression of the early osteogenic differentiation marker runt-related transcription factor 2 (RUNX2) was used to evaluate osteogenesis in cells in the expansion area. After a seven-day RME procedure, RUNX2 expression was increased on the nasal and oral sides of the palatal suture (Figures 1(g) and 1(i)), which was consistent with expression patterns of Gli1^+^ cells. Furthermore, most Gli1^+^ cells showed upregulated expression of RUNX2, indicating that most Gli1^+^ cells participated in mechanical force-induced osteogenesis (Figures 1(g) and 1(j)). These results show that bone remodeling was faster in the RME group and that Gli1^+^ cells residing in maxillofacial sutures participate in mechanical force-induced bone remodeling.

### 3.2. Pharmacological Inhibition of Gli1^+^ Cells Suppresses Mechanical Force-Induced Maxillofacial Bone Remodeling

To further confirm the crucial role of Gli1^+^ cells in mechanical force-induced maxillofacial bone remodeling, *Gli1-LacZ* transgenic mice subjected to RME treatments were treated with GANT61, a small molecular inhibitor of Gli1, to inhibit Gli1^+^ cells (RME+GANT61) while the control group was treated with vehicle only (Figure 2(a)). HE staining showed that the mechanical force-induced cell increase on the nasal and oral sides of the palatal suture was suppressed after the GANT61 treatment, and widening of the palatal suture was also suppressed. Masson's staining showed inhibition of collagen reorienting after GANT61 application (Figure 2(b)). In addition, a decrease in bone deposition rate and lower palatal suture width were observed in the GANT61 treatment group using calcein staining (Figures 2(c) and 2(d)) and micro-CT analysis (Figures 2(e) and 2(f)), respectively. More importantly, immunofluorescence staining demonstrated that GANT61 application prevented the RME-induced increase in Gli1^+^ (Figures 2(g) and 2(h)) and RUNX2^+^ cells (Figures 2(g) and 2(i)) and suppressed RUNX2^+^ and Gli1^+^ cells (Figures 2(g) and 2(j)). Inhibition of Gli1^+^ cells suppressed mechanical force-induced bone remodeling, indicating that Gli1^+^ cells play a critical role in the process of mechanical force-induced bone remodeling.

### 3.3. Mechanical Stretching Induces Gli1^+^ Cell Increases and Regulates JBMMSC Osteogenesis via IP_3_R-Mediated Intracellular Calcium Increases

**Figure 4:**
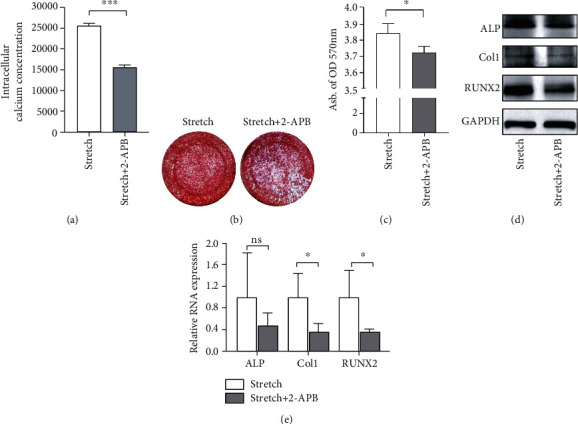
Mechanical stretching regulates JBMMSC osteogenesis via IP_3_R-mediated intracellular calcium increases. (a) Intracellular calcium concentrations visualized at mean fluorescence intensity decreased after 2-APB treatment, compared with the stretch group. ^∗∗∗^*P* < 0.005; *n* = 3. (b) JBMMSCs treated with 2-APB showed fewer mineralized nodules, here stained using alizarin red. (c) The abundance of mineralized nodules in the stretch group was higher than that in the stretch+2-APB group. ^∗^*P* < 0.05; *n* = 3. (d) Western blot analysis indicated downregulation of ALP, RUNX2, and Col1 protein expression in JBMSCs after 2-APB and mechanical stretching treatment, compared with cells subjected only to mechanical stretching. *β*-Tubulin was used as an internal control; *n* = 3. (e) RT-qPCR showed downregulation of ALP, RUNX2, and Col1 RNA expression in JBMSCs after 2-APB and mechanical stretching treatment, compared with cells subjected only to mechanical stretching. GAPDH was used as an internal control. ^∗^*P* < 0.05; ^ns^*P* > 0.05; *n* = 3.

To imitate the mechanical force shared by JBMMSCs during RME progression, mechanical stretching was applied to JBMMSCs separated from the jaw bone of *Gli1-mT/mG* transgenic mice. This type of transgenic mice shows green fluorescence of Gli1^+^ after tamoxifen application, while other cells show red fluorescence. Identification of cells from the jaw bone showed positive expressing certain surface markers of MSCs, such as sca-1, CD29, CD105, and CD73 and negative expressing CD45 and CD11b (Supplementary Figure 3(a)); furthermore, they showed typical MSC including osteogenesis, adipogenesis, and clone formation [8] (Supplementary Figure 3(b)). What is more, flow cytometry showed that Gli1^+^ cells were involved in JBMMSCs under physiological conditions, and the proportion of Gli1^+^ cells increased after mechanical stretching (Figures 3(b) and 3(c)). Moreover, after mechanical stretching, cells were elongated and were rearranged in the direction of the applied mechanical force (Supplementary Figure 4), and it has been reported that elongated MSCs tend to engage in osteogenesis [22]. Thus, JBMMSCs subjected to mechanical stretching and controls were cultured in osteogenic medium, and ALP and alizarin red staining were used to evaluate osteogenic effects in both groups. Both ALP and alizarin red staining showed upregulation of osteogenesis in JBMMSCs after mechanical stretching (Figures 3(d) and 3(e)). Consistently, western blotting and RT-qPCR showed increased expression of osteogenic differentiation markers, including ALP, RUNX2, and Col1 in the mechanical stretch group (Figures 3(f) and 3(g)). Ion channels residing in membrane structures of cells were previously reported to act as mechanical sensors [23, 24], and intracellular calcium ion was recognized as a common second messenger responding to mechanical force [25, 26]. Thus, we measured expression levels of IP_3_R, which is a calcium channel residing in the ER membrane [13, 14]. As expected, RNA and protein expressions of IP_3_R were upregulated after mechanical stretching (Figures 3(f) and 3(h)), and intracellular calcium concentrations were also increased (Figure 3(i)). In order to confirm whether IP_3_R-mediated changes in intracellular calcium concentrations were related to mechanical force-induced JBMMSC osteogenesis, we inhibited IP_3_R using 2-APB, a blocker of IP_3_R, and found that mechanical stretch-induced intracellular calcium was reduced (Figure 4(a)) and that mechanical stretch-induced osteogenesis was also inhibited (Figures 4(b)–4(e)). In summary, mechanical stretching increased the proportion of Gli1^+^ cells and induced JBMMSC osteogenesis through an IP_3_R-mediated increase in intracellular calcium concentrations. Based on these results, we speculate that Gli1^+^ cells may participate in mechanical force-mediated JBMMSC osteogenesis through an IP_3_R-mediated increase in intracellular calcium concentrations.

### 3.4. Gli1^+^ Cells Participate in Mechanical Force-Mediated JBMMSC Osteogenesis through an IP_3_R-Induced Intracellular Calcium Concentration Increase

**Figure 5:**
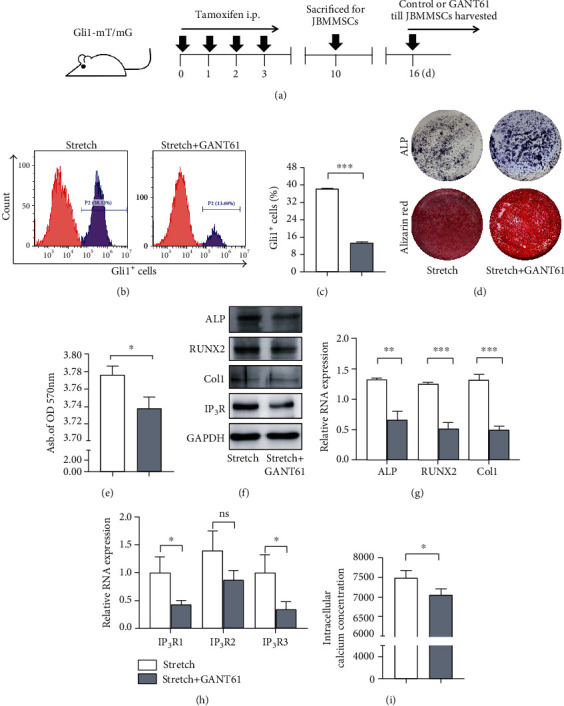
Gli1^+^ cells participate in mechanical force-mediated JBMMSC osteogenesis through an IP_3_R-induced intracellular calcium concentration increase. (a) Experimental procedure: *Gli1-mT/mG* mice were treated with tamoxifen for 4 consecutive days and were sacrificed after 7 days to isolate JBMMSCs. After 6 days of incubation, cells were treated with GANT61 (stretch+GANT61) or vehicle (stretch). (b) Flow cytometry analysis of the proportion of Gli1^+^ cells in JBMMSCs showed a decrease of Gli1^+^ cells in the GANT61-treatment; *n* = 3. (c) The proportion of Gli1^+^ cells was lower in the stretch+GANT61 group; ^∗∗∗^*P* < 0.005; *n* = 3. (d) ALP activity of JBMMSCs treated with GANT61 was reduced after osteogenic induction for 7 days. Mineralized nodules formed by JBMMSCs were detected using alizarin red staining after osteogenic induction for 14 days. (e) The abundance of mineralized nodules in the stretch+GANT61 group was lower; ^∗^*P* < 0.05; *n* = 3. (f) Western blot analysis showed downregulation of ALP, RUNX2, Col1, and IP_3_R protein expression in JBMMSCs treated with GANT61 and mechanically stretched, compared with cells subjected only to mechanical stretching; *n* = 3. (g) RT-qPCR showed downregulation of ALP, RUNX2, and Col1 RNA expression in JBMMSCs treated with GANT61 and mechanically stretched, compared with cells subjected only to mechanical stretching. ^∗∗∗^*P* < 0.005; ^∗∗^*P* < 0.01; *n* = 3. (h) RT-qPCR showed downregulation of IP_3_R1, IP_3_R2, and IP_3_R3 RNA expression in JBMMSCs treated with mechanical stretching and GANT61, compared with cells subjected only to mechanical stretching; ^∗^*P* < 0.05; ^ns^*P* > 0.05; *n* = 3. (i) Intracellular calcium concentration visualized at mean fluorescence intensity was decreased in the stretch+GANT61 group. ^∗^*P* < 0.05; *n* = 3.

To confirm whether Gli1^+^ cells involved in mechanical stretching induced JBMMSC osteogenesis, we pharmacologically inhibited Gli1^+^ cells in JBMMSCs using GANT61. Six days after JBMMSCs were isolated from *Gli1-mT/mG* transgenic mice, GANT61 was applied until JBMMSCs were harvested for the follow-up experiments (Figure 5(a)). Flow cytometry demonstrated that the mechanical stretching-induced increase in Gli1^+^ cells was inhibited by GANT61 (Figures 5(b) and 5(c)). ALP and alizarin red staining showed an inhibition of osteogenesis in the Gli1^+^ cell inhibition group (Figures 5(d) and 5(e)), and western blotting and RT-qPCR demonstrated downregulation of ALP, RUNX2, and Col1 in the GANT61 group (Figures 5(f) and 5(g)). These results were consistent with the in vivo experiment and confirmed that Gli1^+^ cells play an important role in mechanical stretch-induced osteogenesis of JBMMSCs. In line with our hypothesis, the upregulated RNA and protein expression of IP_3_R induced by mechanical stretching was downregulated due to inhibition of Gli1^+^ cells (Figures 5(f) and 5(h)), which also led to reduced intracellular calcium concentrations (Figure 5(i)) and thus reduced the osteogenic potential of JBMMSCs. Taken together, our results suggest that Gli1^+^ cells participate in mechanical force-mediated JBMMSC osteogenesis through an IP_3_R-mediated increase in intracellular calcium concentrations.

### 3.5. Gli1^+^ Cells Participate in RME-Induced Bone Formation via IP_3_R Upregulation

**Figure 6:**
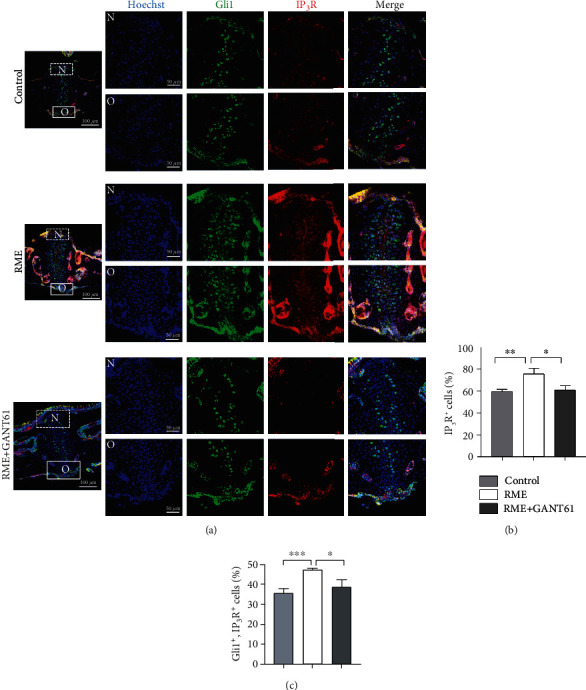
Gli1^+^ cells participate in RME-induced bone formation via IP_3_R upregulation. (a) Distribution of IP_3_R^+^ cells (red), Gli1^+^ cells (green), and IP_3_R^+^ and Gli1^+^ cells in the control, RME, and RME+GANT61 groups detected with immunofluorescence staining. Scale bar: 100 *μ*m. Regions in boxes are magnified in the right panel: “N” indicates the nasal side of the midpalatal suture and “O” indicates the oral side. Scale bar: 50 *μ*m; *n* = 3. (b) IP_3_R^+^ cells were increased in the RME group and decreased after GANT61 treatment. ^∗^*P* < 0.05; ^∗∗^*P* < 0.01; *n* = 3. (c) IP_3_R^+^ and Gli1^+^ cells increased in the RME group and decreased after GANT61 treatment. ^∗∗∗^*P* < 0.005; ^∗^*P* < 0.05; *n* = 3.

To confirm the effects of Gli1^+^ cells on mechanical force-induced IP_3_R upregulation, we measured IP_3_R^+^ cells and Gli1^+^ cells in palatal suture of RME mouse models to support in vitro experiments. And found that IP_3_R^+^ cells increased in RME mice compared with the controls (Figures 6(a) and 6(b)), and most of the increased IP_3_R^+^ cells were Gli1^+^ cells (Figures 6(a) and 6(c)). After inhibition of Gli1^+^ cells in RME mice, mechanical force-induced IP_3_R^+^ cells increase impeded as well (Figures 6(a) and 6(b)), and so did Gli1^+^ and IP_3_R^+^ cells (Figures 6(a) and 6(c)). The in vivo experiment thus also demonstrated that Gli1^+^ cells participated in mechanical force-induced bone formation through IP_3_R upregulation.

## 4. Discussion

Early orthodontic treatment of skeletal malocclusion relies on unclosed sutures in the maxillofacial bones [27]; however, it remains unclear how cells residing in sutures react to mechanical force. Gli1^+^ cells in maxillofacial sutures are the basis of maxillofacial bone development and damage repair [10], and Gli1^+^ periodontium stem cells have been identified as mechanical sensors during mechanical force-induced alveolar bone remodeling [12, 28]. In the current study, Gli1^+^ cells residing in maxillofacial sutures were found to participate in mechanical force-induced bone formation through an IP_3_R-mediated increase in intracellular calcium levels in vitro and in vivo.

Gli1^+^ cells are recognized as a subpopulation of MSCs residing surrounding neurovascular bundle (NVB) of dental pulp and bones and are crucial for osteogenesis and odontogenesis under physiological and pathological conditions [9, 29]. Recently, these cells were found to reside in proximity of the NVB of the periodontium and at the front of bone and cementum formation where they act as mechanical sensors during mechanical force-induced bone remodeling [12, 28, 30]. Unlike long bones, maxillofacial bones are flat bones with little bone marrow and few NVBs [31, 32]; however, Gli1^+^ cells are still found in the periosteum, the dura, and in the suture mesenchyme of maxillofacial bones, and are able to participate in bone formation [10]. And as predicted, Gli1^+^ cells residing in the maxillofacial bone were found to act as mechanical sensors after stimulation by mechanical force. After pharmacological inhibition of Gli1 expression, mechanical force-induced bone remodeling was impeded. This is consistent with the clinical finding that RME treatment shows no expansion effect on midpalatal sutures in patients with solitary median maxillary central incisor syndrome (SMMCI), which was proven to be caused by a mutation in the *SHH* gene upstream of *Gli1* [33]. Thus, there is reasonable doubt about whether Gli1^+^ cell deficiency occurs in patients with SMMCI, and our study provides valuable insights which may help understand this particular syndrome.

So far, Gli1^+^ cells have only been shown to act as mechanical sensors in mechanical force-induced bone remodeling progress in vivo. Thus, we performed an in vitro experiment by applying mechanical stretching to JBMMSCs to assess changes in the Gli1^+^ cell population and in JBMMSC osteogenesis. Since cells residing in suture were very limited and Gli1^+^ cells are a considerably small population of MSCs under physiological conditions [10, 34], thus it is complicated to acquire MSCs from midpalatal sutures and to then isolate Gli1^+^ cells from them. Our in vivo experiments showed that Gli1^+^ cells increased not only at the centre of the suture but also in the periosteum around the suture. We therefore separated cells from the jaw bone and examined mechanosensing functions of Gli1^+^ cells during mechanical force-induced osteogenesis by downregulating Gli1 expression. Cells separated from the jaw bone showed typical MSC characteristics, and when cells were cultured to passage 3, the proportion of Gli1^+^ cells was about 30%, which is in line with previous research showing that Gli1^+^ cells residing in maxillofacial suture were MSCs [10]. Our findings imply that Gli1^+^ cells examined in the current study are in fact MSCs. Because of technical limitations, it was difficult for us to separate Gli1^+^ cells and to directly examine their characteristics under physiological and during mechanical forcing. Thus, more effort should be made to resolve such technical limitations.

Molecules located on cell membranes can sense mechanical signals and transduce them to the nucleus [35, 36]. And calcium ion channels located on cell membranes play a vital role in mechanical force transduction [24]. IP_3_R which is a type of calcium ion channels mainly located on ER membrane is related to mechanical force-induced calcium change in MSCs [25]. In the present study, we observed that IP_3_R responded to mechanical force and elicited changes in intracellular calcium concentrations during mechanical force-induced bone remodeling. To our knowledge, our study is the first to describe the association between Gli1^+^ cells and IP_3_R-mediated intracellular calcium concentration changes during mechanical force-induced bone formation. However, our results only provide preliminary insights into how Gli1^+^ cells may regulate mechanical force-induced osteogenesis. The mechanism by which Gli1 regulates IP_3_R and how intracellular calcium concentration changes affect osteogenesis after mechanical force application require further research. Besides, since 2-APB is mainly used in vitro experiment, and it is hard for mouse to survive after multiple drug treatment and RME operation, we only blocked the function of IP_3_R in vitro experiment to verify the crucial role of IP_3_R in mechanical force-induced osteogenesis; more effort should be made to conquer technical limitation to regulate IP_3_R in vivo and confirm the role of IP_3_R in mechanical force-induced bone formation during RME process.

## 5. Conclusions

We demonstrated the crucial role of Gli1^+^ cells residing in maxillofacial sutures during mechanical force-induced bone remodeling using in vivo and in vitro experiments. Gli1^+^ cells participated in mechanical force-mediated osteogenesis by regulating IP_3_R-mediated intracellular calcium concentration, suggesting a novel approach for the orthodontic treatment of skeletal malocclusion.

## Figures and Tables

**Table 1:** Primers used for gene typing.

Gene type	Forward primer sequence (5′–3′)	Reverse primer sequence (5′–3′)
*Gli1-LacZ* (wild type)	GGGATCTGTGCCTGAAACTG	AGGTGAGACGACTGCCAAGT
*Gli1-LacZ* (mutant type)	GGGATCTGTGCCTGAAACTG	TCTGCCAGTTTGAGGGGACGAC
*Gli1-CreER^T2^* (wild type)	GCGGTCTGGCAGTAAAAACTATC	GTGAAACAGCATTGCTGTCACTT
*Gli1-CreER^T2^* (mutant type)	GGGATCTGTGCCTGAAACTG	CTTGTGGTGGAGTCATTGGA
*mT/mG* (wild type)	CTCTGCTGCCTCCTGGCTTCT	CGAGGCGGATCACAAGCAATA
*mT/mG* (mutant type)	CTCTGCTGCCTCCTGGCTTCT	TCAATGGGCGGGGGTCGTT

**Table 2:** Antibodies used in this research.

Antibody	Manufacture	Catalogue number	Concentration	Application
Anti-GAPDH	Yesen Biotechnology	30201ES20	1 : 2000	WB
Anti-beta tubulin	Proteintech	10068-1-AP	1 : 2000	WB
Anti-RUNX2	Cell Signaling Technology	12556	1 : 200 for IF; 1 : 1000 for WB	IF; WB
Anti-*β*-gal	Abcam	ab9361	1 : 200	IF
Anti-IP_3_R	Abcam	ab108517	1 : 200 for IF; 1 : 1000 for WB	IF; WB
Anti-ALP	Santa Cruz Biotechnology	sc-79840	1 : 1000	WB
Anti-Col1	Abcam	ab34710	1 : 1000	WB
Anti-CD73	eBioscience	12-0731	1 : 100	FC
Anti-CD105	BioLegend	120413	1 : 100	FC
Anti-CD29	eBioscience	17-0291-82	1 : 100	FC
Anti-CD11b	BioLegend	101227	1 : 100	FC
Anti-CD45	BD Pharmingen	553134	1 : 100	FC
Anti-sca-1	eBioscience	17-5981	1 : 100	FC

**Table 3:** Primers used for RT-qPCR.

Gene	Forward primer sequence (5′–3′)	Reverse primer sequence (5′–3′)
GAPDH	TGTGTCCGTCGTGGATCTGA	TTGCTGTTGAAGTCGCAGGAG
ALP	CCAACTCTTTTGTGCCAGAGA	GGCTACATTGGTGTTGAGCTT TT
RUNX2	GACTGTGGTTACCGTCATGGC	ACTTGGTTTTTCATAACAGCGGA
Col1	GCTGGAGTTTCCGTGCCT	GACCTCGGGGACCCATTG
IP_3_R1	ATTTGTTCTCTGTATGCGGAGG	AGCTTAAAGAGGCAGTCTCTGA
IP_3_R2	CCTCGCCTACCACATCACC	TCACCACTCTCACTATGTCGT
IP_3_R3	GCCCTTACATGCCAGCAACTA	GCTTGCCCCTGTACTCATCAC

## Data Availability

The raw data used to support the findings of this study are available from the corresponding authors upon request.

## Supplementary Materials

Supplementary Materials Supplementary Figure 1: (a) intraoral view of a RME mouse model and (b) occlusal view of the mouse maxilla. RME maxilla with the opening loop fixed to the molars (left) and untreated control (right). Supplementary Figure 2: (a) HE staining of the coronal plane of the maxilla at different time points (days 0, 1, 3, 7, and 28) and (b) distribution of Gli1^+^ cells (green) in midpalatal sutures at different time points of RME (days 0, 1, 7, and 14) as detected by immunofluorescence staining. Supplementary Figure 3: (a) surface markers of JBMMSCs analyzed using flow cytometry (JBMMSCs positively express CD29, sca-1, CD73, and CD105 and negatively express CD11b and CD45), (b) alizarin red staining after osteogenic induction of JBMMSs demonstrates mineralized nodules formed by JBMMSCs, (c) Oil Red O staining after adipogenic induction of JBMMSCs showed lipid restoration in JBMMSCs, and (d) crystal violet staining of colony formed by JBMMSCs. Supplementary Figure 4: (a) static state of JBMMSCs and (b) JBMMSCs under mechanical stretching.
