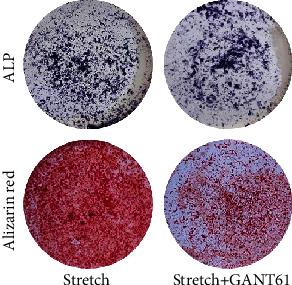# Erratum to “Gli1+ Cells Residing in Bone Sutures Respond to Mechanical Force via IP3R to Mediate Osteogenesis”

**DOI:** 10.1155/2021/9897280

**Published:** 2021-11-20

**Authors:** Xiaoyao Huang, Zihan Li, Peisheng Liu, Meiling Wu, An-qi Liu, Chenghu Hu, Xuemei Liu, Hao Guo, Xiaoxue Yang, Xiaohe Guo, Bei Li, Xiaoning He, Kun Xuan, Yan Jin

**Affiliations:** ^1^State Key Laboratory of Military Stomatology & National Clinical Research Center for Oral Diseases & Shaanxi Clinical Research Center for Oral Diseases, Department of Preventive Dentistry, School of Stomatology, The Fourth Military Medical University, Xi'an 710032, China; ^2^State Key Laboratory of Military Stomatology & National Clinical Research Center for Oral Diseases & Shaanxi International Joint Research Center for Oral Diseases, Center for Tissue Engineering, School of Stomatology, The Fourth Military Medical University, Xi'an 710032, China; ^3^College of Life Science, Northwest University, Xi'an, China

In the article titled, “Gli1+ Cells Residing in Bone Sutures Respond to Mechanical Force via IP3R to Mediate Osteogenesis” [1], there is an error in Figures 3(d) and 5(d) which was introduced during production process. The corrected Figures 3(d) and 5(d) is shown below.

## Figures and Tables

**Figure 1:**
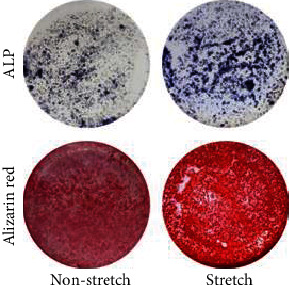


**Figure 2:**