# Supplementary material for: Gli1+ Cells Residing in Bone Sutures Respond to Mechanical Force via IP3R to Mediate Osteogenesis
Source: Stem Cells Int. 2021 Aug 12;2021:8138374. doi: 10.1155/2021/8138374 (PMC8380501; doi:10.1155/2021/8138374)
Supplement: Supplementary Materials — Supplementary Figure 1: (a) intraoral view of a RME mouse model and (b) occlusal view of the mouse maxilla. RME maxilla with the opening loop fixed to the molars (left) and untreated control (right). Supplementary Figure 2: (a) HE staining of the coronal plane of the maxilla at different time points (days 0, 1, 3, 7, and 28) and (b) distribution of Gli1+ cells (green) in midpalatal sutures at different time points of RME (days 0, 1, 7, and 14) as detected by immunofluorescence staining. Supplementary Figure 3: (a) surface markers of JBMMSCs analyzed using flow cytometry (JBMMSCs positively express CD29, sca-1, CD73, and CD105 and negatively express CD11b and CD45), (b) alizarin red staining after osteogenic induction of JBMMSs demonstrates mineralized nodules formed by JBMMSCs, (c) Oil Red O staining after adipogenic induction of JBMMSCs showed lipid restoration in JBMMSCs, and (d) crystal violet staining of colony formed by JBMMSCs. Supplementary Figure 4: (a) static state of JBMMSCs and (b) JBMMSCs under mechanical stretching. [file 8138374.f1.docx]

**Supplementary Material**

**
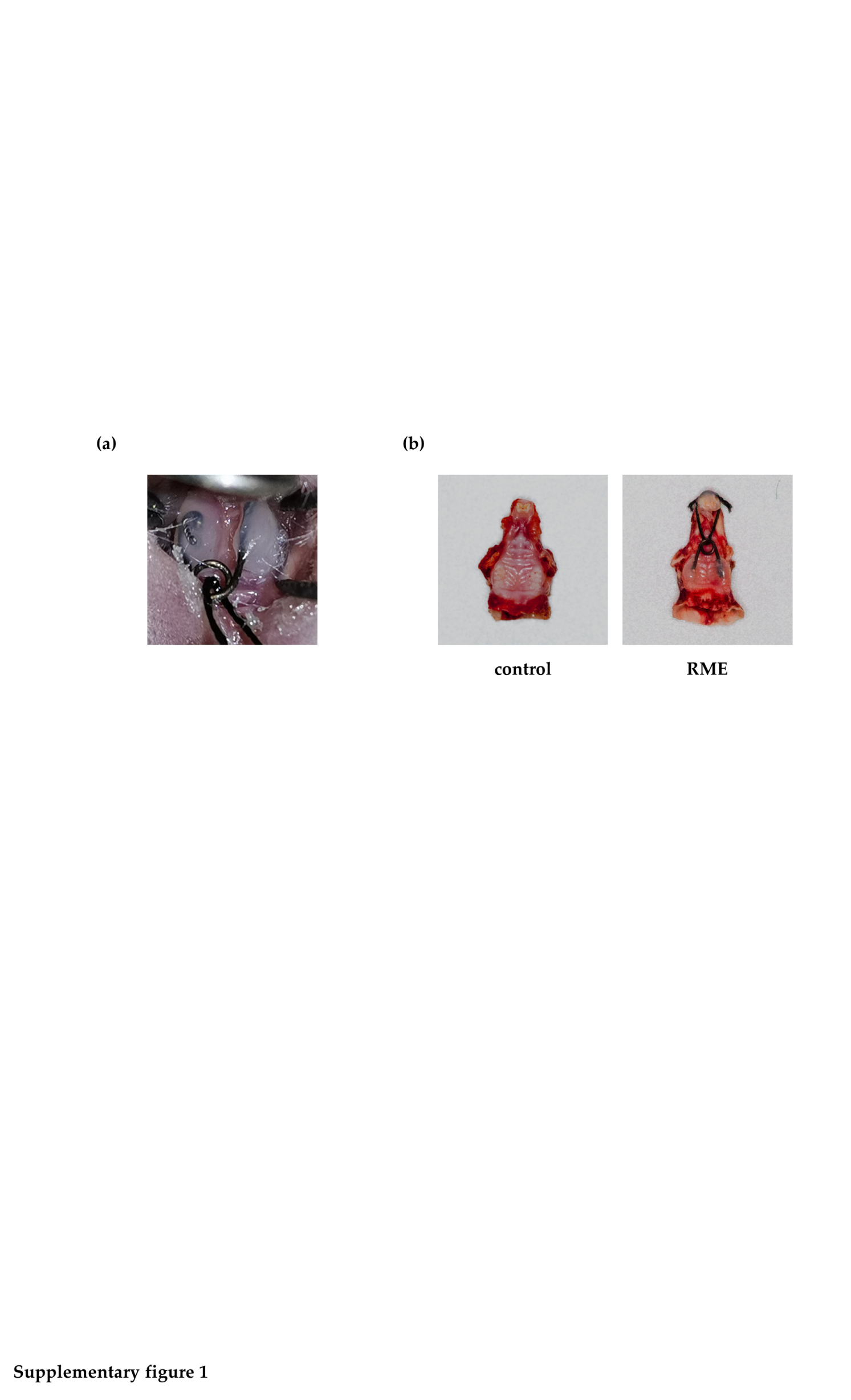
**

**Supplementary Figure 1.** (a), Intraoral view of a RME mouse model. (b), Occlusal view of the mouse maxilla. RME maxilla with the opening loop fixed to the molars (left) and untreated control (right).


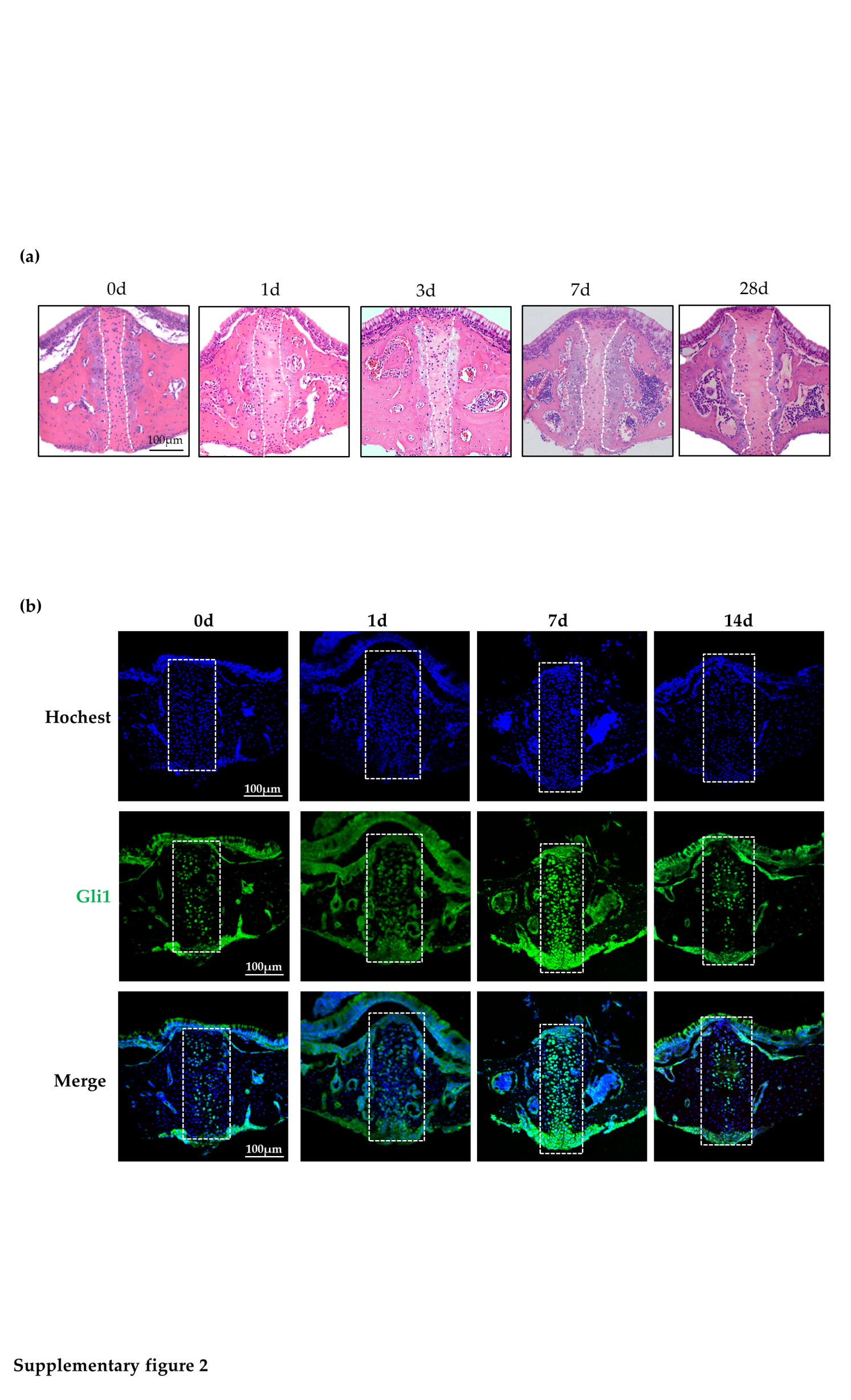


**Supplementary Figure 2.** (a), HE staining of the coronal plane of the maxilla at different time points (days 0, 1, 3, 7, and 28). (b), Distribution of Gli1^+^ cells (green) in mid-palatal sutures at different time points of RME (days 0, 1, 7, and 14) as detected by immunofluorescence staining.


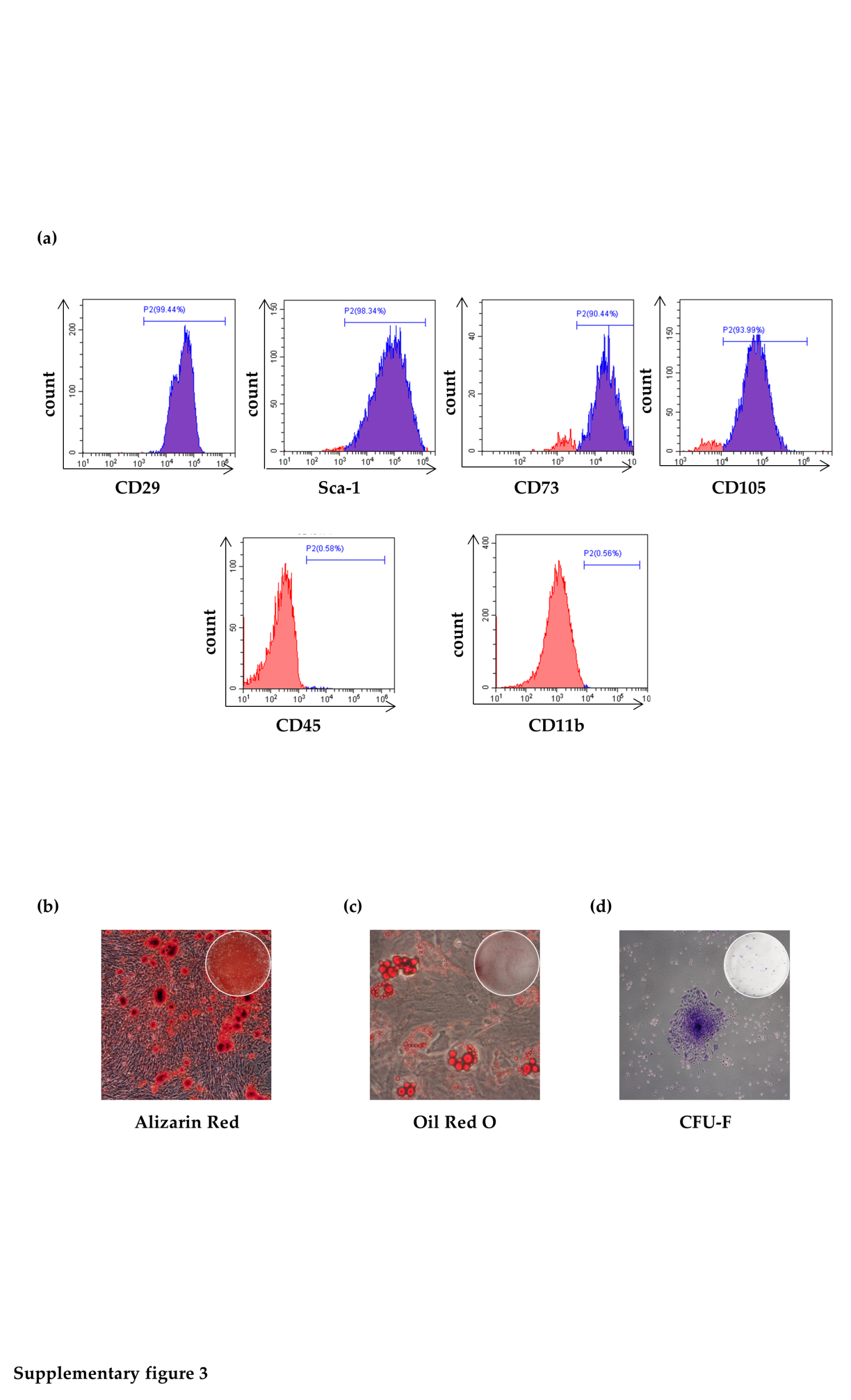


**Supplementary Figure 3.** **(**a), Surface markers of JBMMSCs analyzed using flow cytometry; JBMMSCs positive express CD29, sca-1, CD73 and CD105, and negative express CD11b and CD45. (b), Alizarin red staining after osteogenic induction of JBMMSs demonstrates mineralized nodules formed by JBMMSCs. (c), Oil Red O staining after adipogenic induction of JBMMSCs showed lipid restoration in JBMMSCs. (d), Crystal violet staining of colony formed by JBMMSCs.


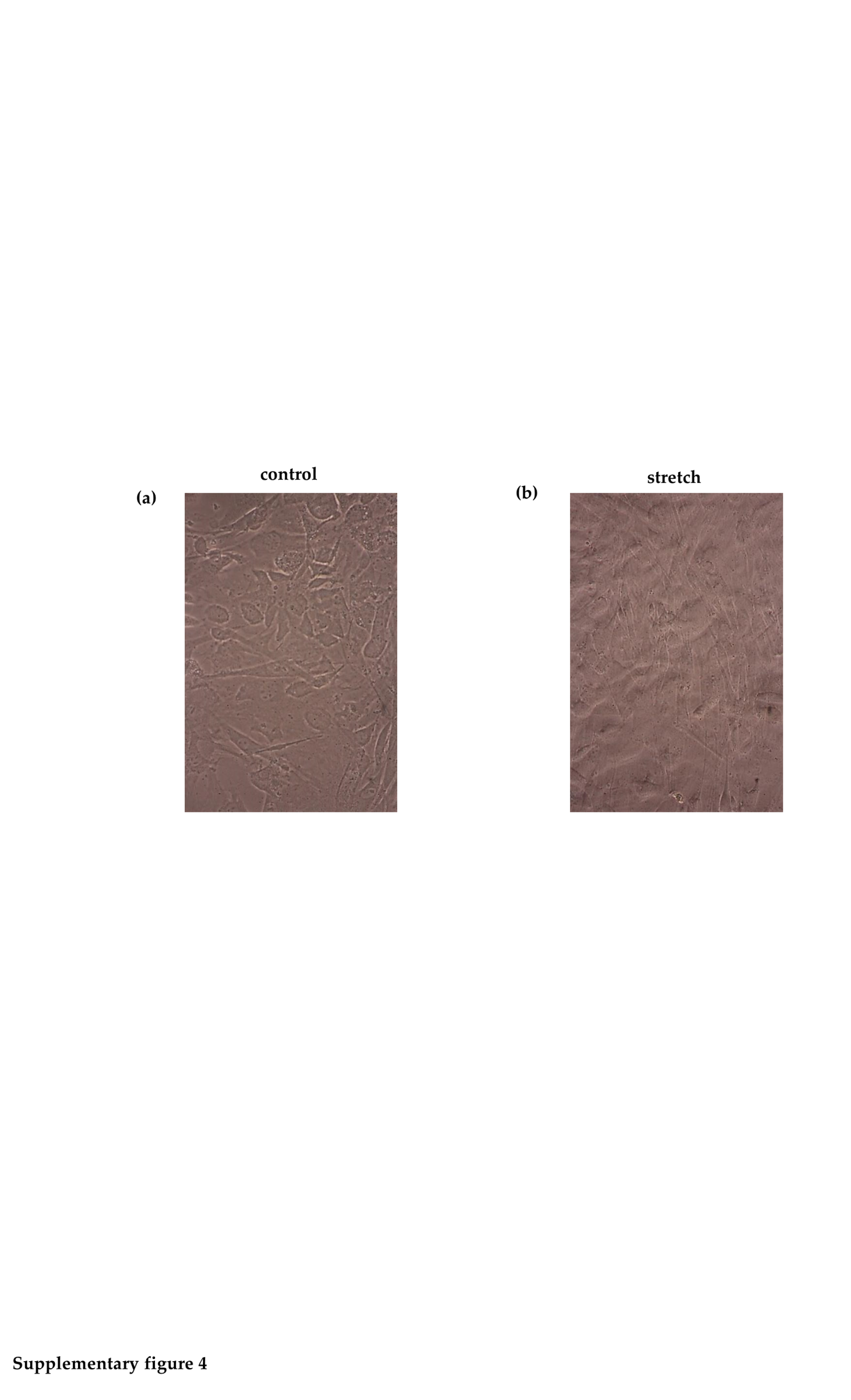


**Supplementary Figure 4.** (a), Static state of JBMMSCs; (b), JBMMSCs under mechanical stretching.
